# Supplementary material for: An antibonding valence band maximum enables defect-tolerant and stable GeSe photovoltaics
Source: Nat Commun. 2021 Jan 28;12:670. doi: 10.1038/s41467-021-20955-5 (PMC7844217; doi:10.1038/s41467-021-20955-5)
Supplement: Supplementary file 1 — Supplementary Information [file 41467_2021_20955_MOESM1_ESM.pdf]

# Supplementary Information for

## An antibonding valence band maximum enables defect-tolerant and stable GeSe photovoltaics

Shun-Chang Liu[1,2†], Chen-Min Dai[3†], Yimeng Min[4], Yi Hou[4], Andrew H. Proppe[4], Ying Zhou[5], Chao Chen[5], Shiyou Chen[3], Jiang Tang[5], Ding-Jiang Xue[1,2*], Edward H. Sargent[4*] and Jin-Song Hu[1,2*]

[1]Beijing National Laboratory for Molecular Sciences (BNLMS), CAS Key Laboratory of Molecular Nanostructure and Nanotechnology, Institute of Chemistry, Chinese Academy of Sciences, Beijing 100190, China

[2]University of Chinese Academy of Sciences, Beijing 100049, China

[3]Key Laboratory of Polar Materials and Devices (MOE), East China Normal University, Shanghai 200241, China

[4]Department of Electrical and Computer Engineering, University of Toronto, Toronto, Ontario M5S 1A4, Canada

[5]Wuhan National Laboratory for Optoelectronics (WNLO), Huazhong University of Science and Technology, Wuhan 430074, China

[†]These authors contributed equally to this work: Shun-Chang Liu, Chen-Min Dai

*e-mail: djxue@iccas.ac.cn; ted.sargent@utoronto.ca; hujs@iccas.ac.cn

This file includes Supplementary Figure 1 to 15 and Supplementary Table 1.

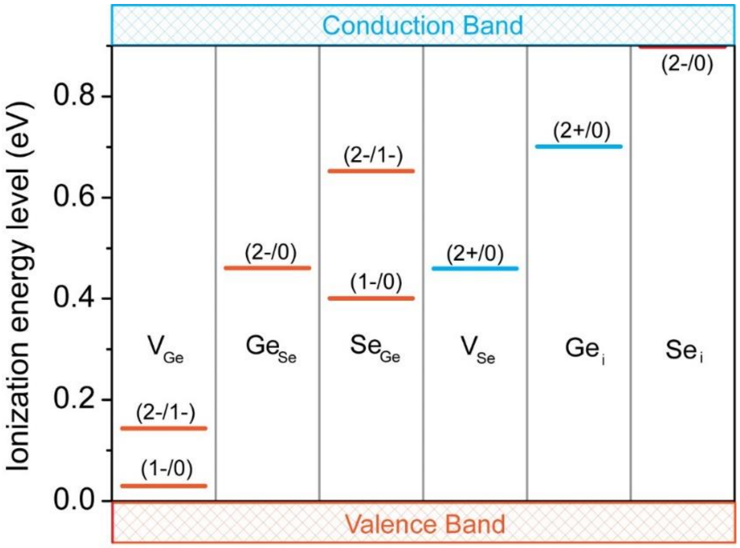


**Supplementary Figure 1 | The calculated ionization levels of intrinsic defects in the band gap of GeSe.** The red bars show the acceptor levels, and the blue bars represent the donor levels. The initial and final charge states are labeled in parentheses.

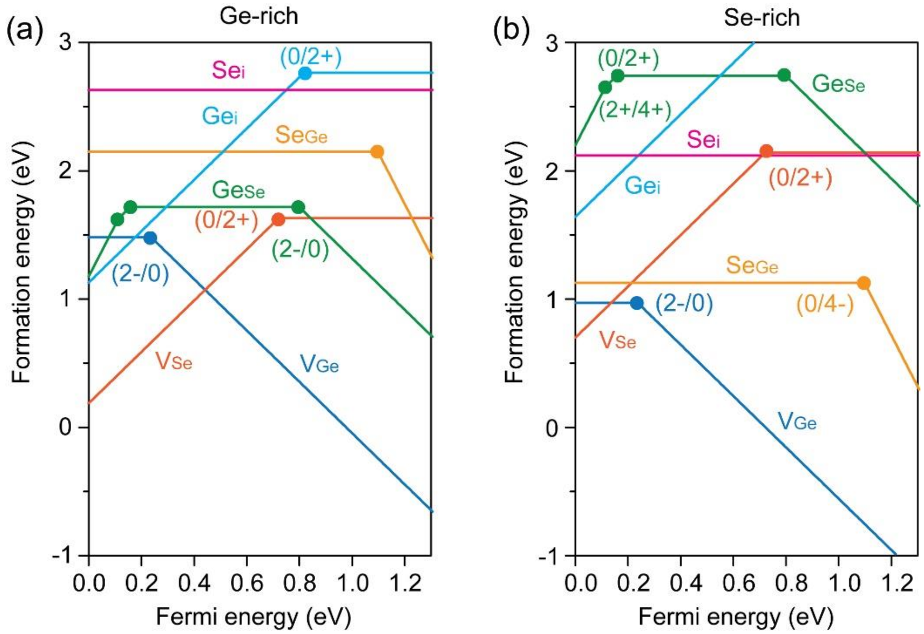


**Supplementary Figure 2 | Point defect properties of GeSe.** Calculated formation energies of intrinsic point defects in GeSe under (a) Ge-rich and (b) Se-rich conditions as a function of the Fermi energy using the Heyd-Scuseria-Ernzerhof (HSE).

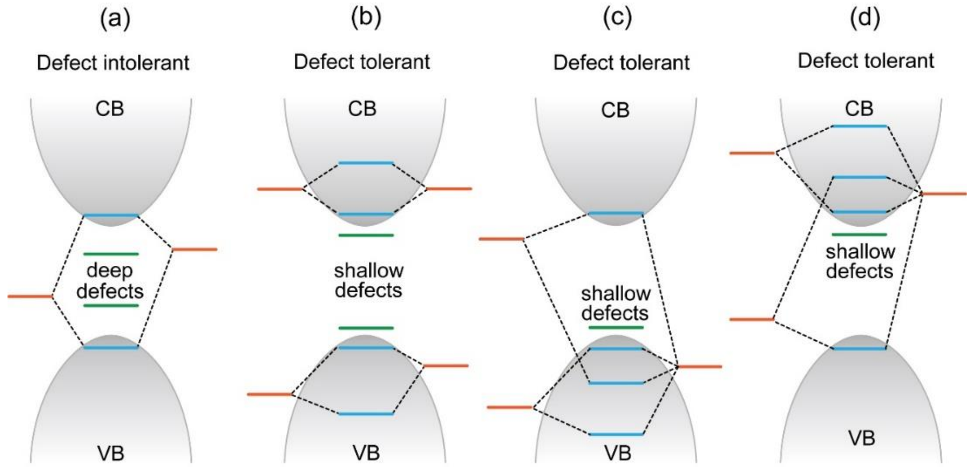


**Supplementary Figure 3 | Electronic structures of different types of semiconductors.** (a) Electronic structure of defect-intolerant semiconductors. (b) Electronic structure of defect-tolerant semiconductors for both acceptor and donor defects. (c) Electronic structure of defect-tolerant semiconductors for acceptor defects. (d) Electronic structure of defect-tolerant semiconductors for donor defects.

Generally, the majority of semiconducting materials possess the bonding VBM and antibonding CBM (Supplementary Fig. 3a). For example, the conduction band of conventional photovoltaic material of CdTe consists of antibonding states of Cd s- and Te p-orbitals[1]. This kind of electronic structure leads to deep defects, which is called defect intolerance[2]. In addition, there are also three types of defect-tolerant electronic structures of semiconductors that result in shallow defects: i) an electronic structure with the antibonding character at VBM and bonding character at CBM (Supplementary Fig. 3b). In this structure, both acceptor and donor defects are shallow[3,4]; ii) an electronic structure only with the antibonding character at VBM (Supplementary Fig. 3c). In this structure, only acceptor defects are shallow[5,6]; iii) an electronic structure only with the bonding character at CBM (Supplementary Fig. 3d). Only donor acceptor defects are shallow in this structure[7].

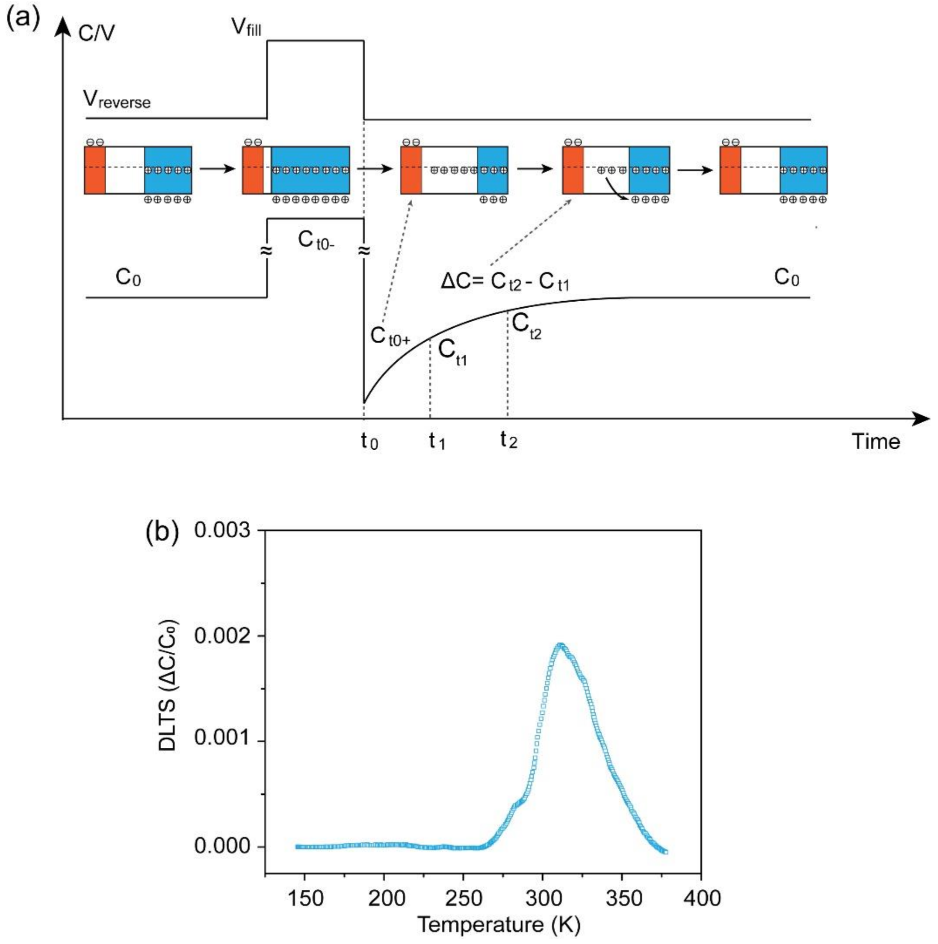


**Supplementary Figure 4 | Deep-level transient spectroscopy (DLTS) characterization.** (a) Schematic of the mechanism of DLTS measurement and the corresponding process of holes being trapped and emitted during the measurement. (b) DLTS signals of the device using a charging voltage of 0.9 V in the range of 145-380 K. $C_{t0+}$ is the junction capacitance at the moment after pulse voltage ended.

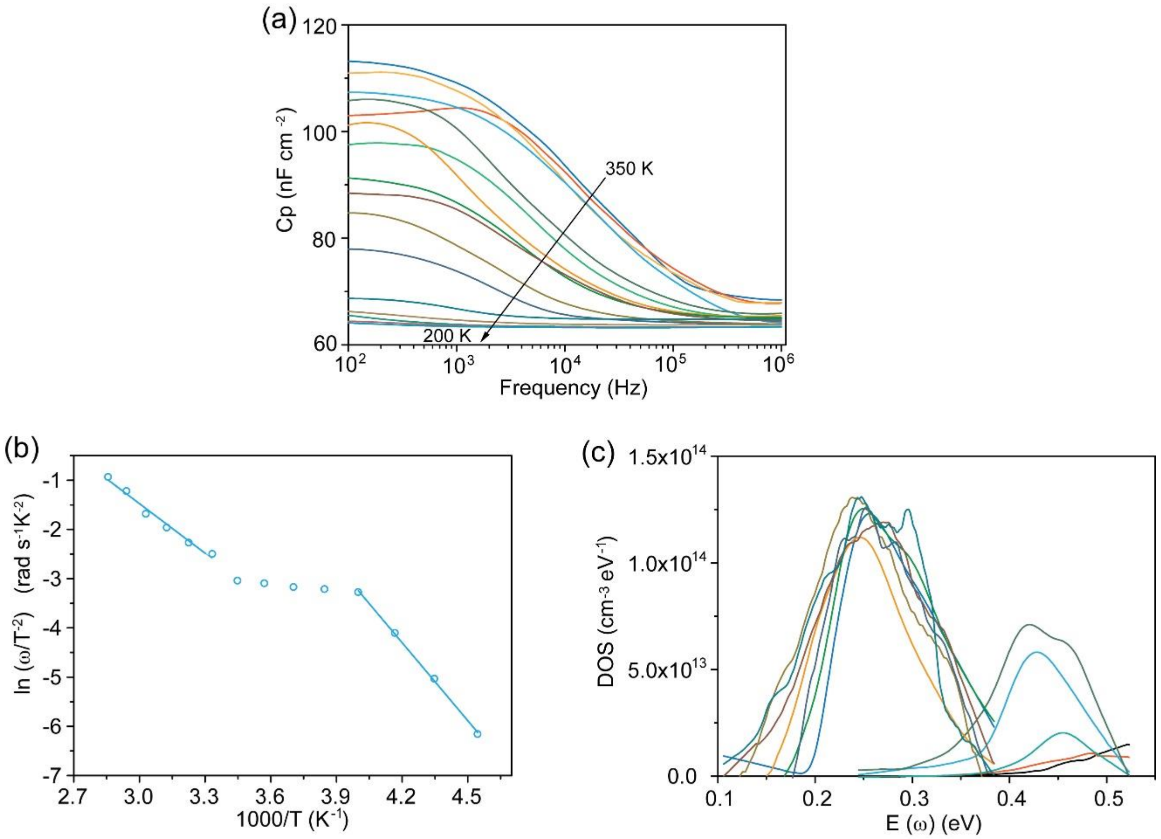


**Supplementary Figure 5 | Admittance spectroscopy (AS) characterization.** (a) The temperature-dependent admittance spectra at temperature between 200 K and 350 K with a step of 10 K. (b) Arrhenius plots determined from the derivative of the admittance spectra. (c) Defect spectra of GeSe solar cell derived from the admittance spectra. The energy peaks indicate two defects levels.

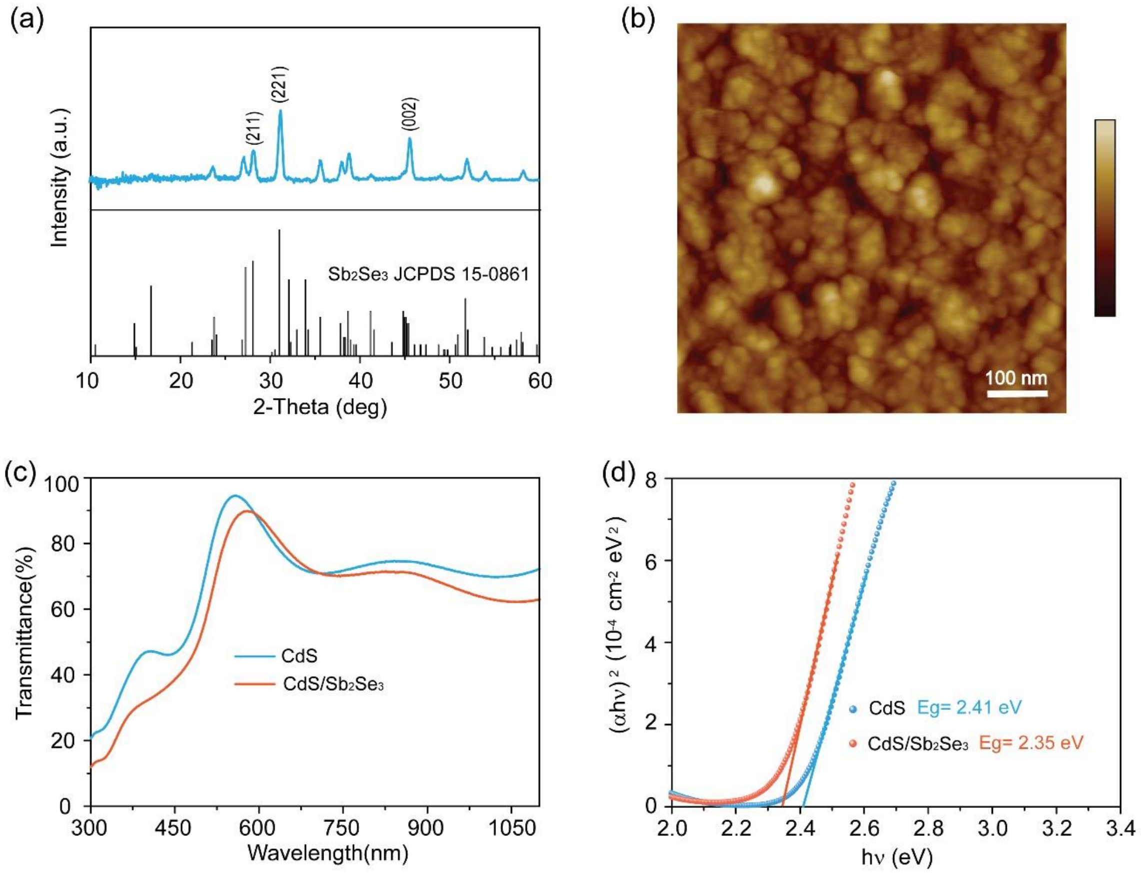


**Supplementary Figure 6 | Characterization of CdS and $Sb_2Se_3$ films.** (a) Grazing incidence XRD pattern of $Sb_2Se_3$ film. (b) AFM image of CdS film. (c) Transmittance spectra of CdS and CdS/$Sb_2Se_3$ films. (d) Tauc plots for CdS and CdS/$Sb_2Se_3$ films.

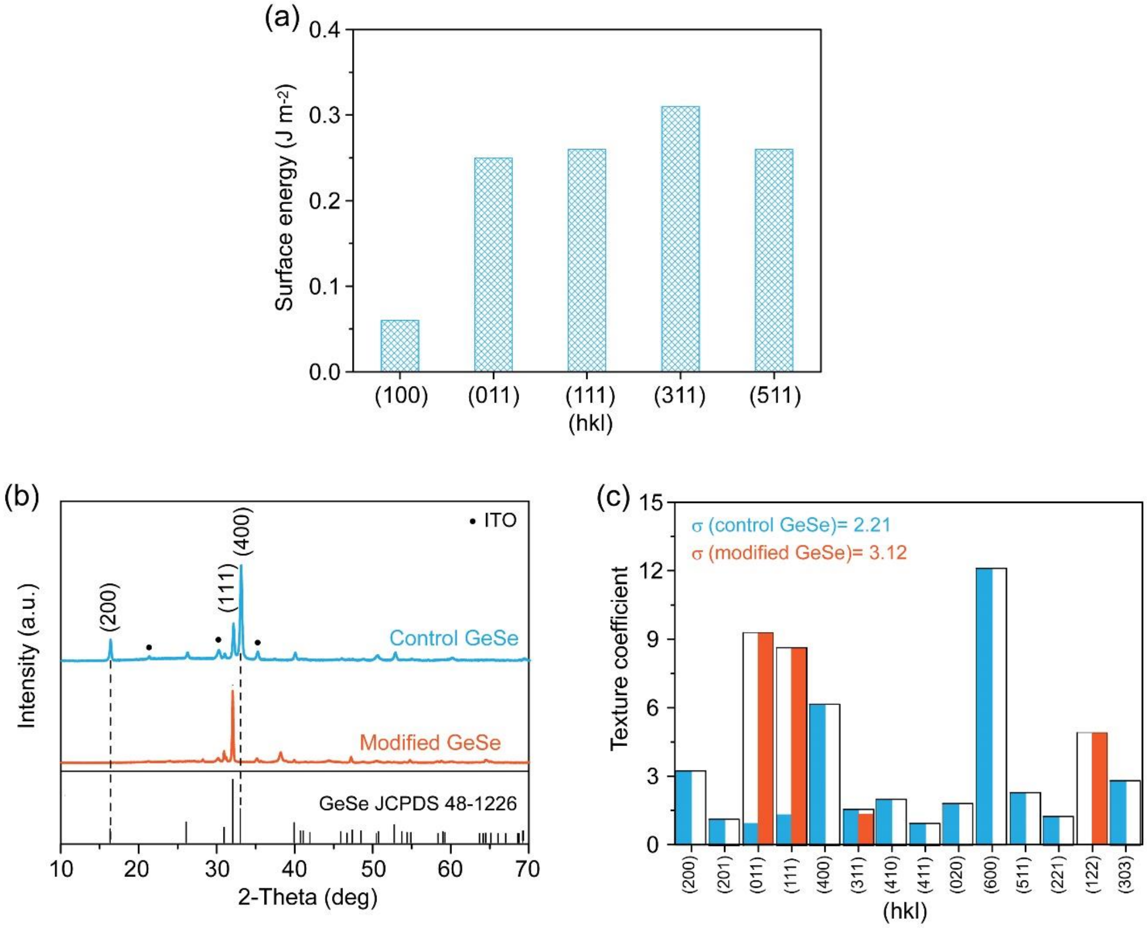


**Supplementary Figure 7 | Characterization of GeSe films.** (a) Calculated surface energies for different surfaces of GeSe. (b) XRD patterns of control and modified GeSe films. (c) Texture coefficients of control and modified GeSe films.

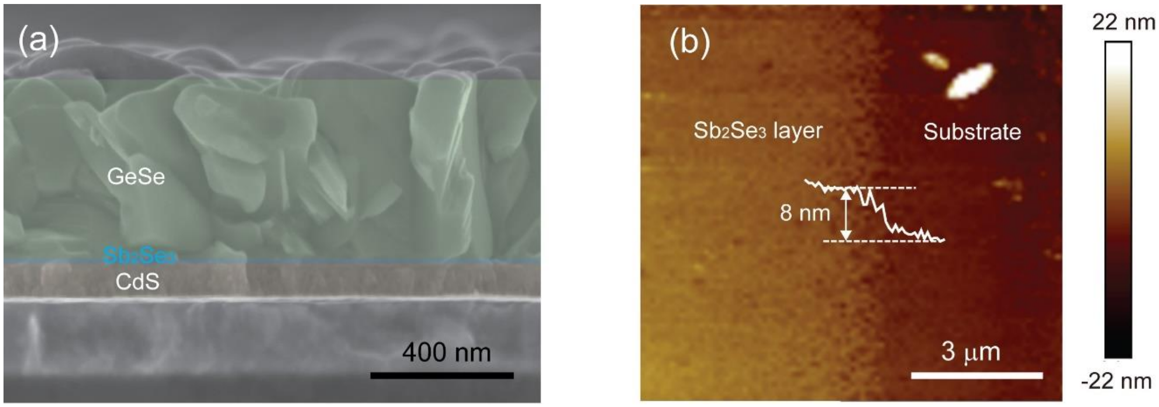


**Supplementary Figure 8 | Characterization of different functional layers.** (a) Cross-sectional SEM image of the GeSe solar cell. (b) AFM image of $Sb_2Se_3$ layer.

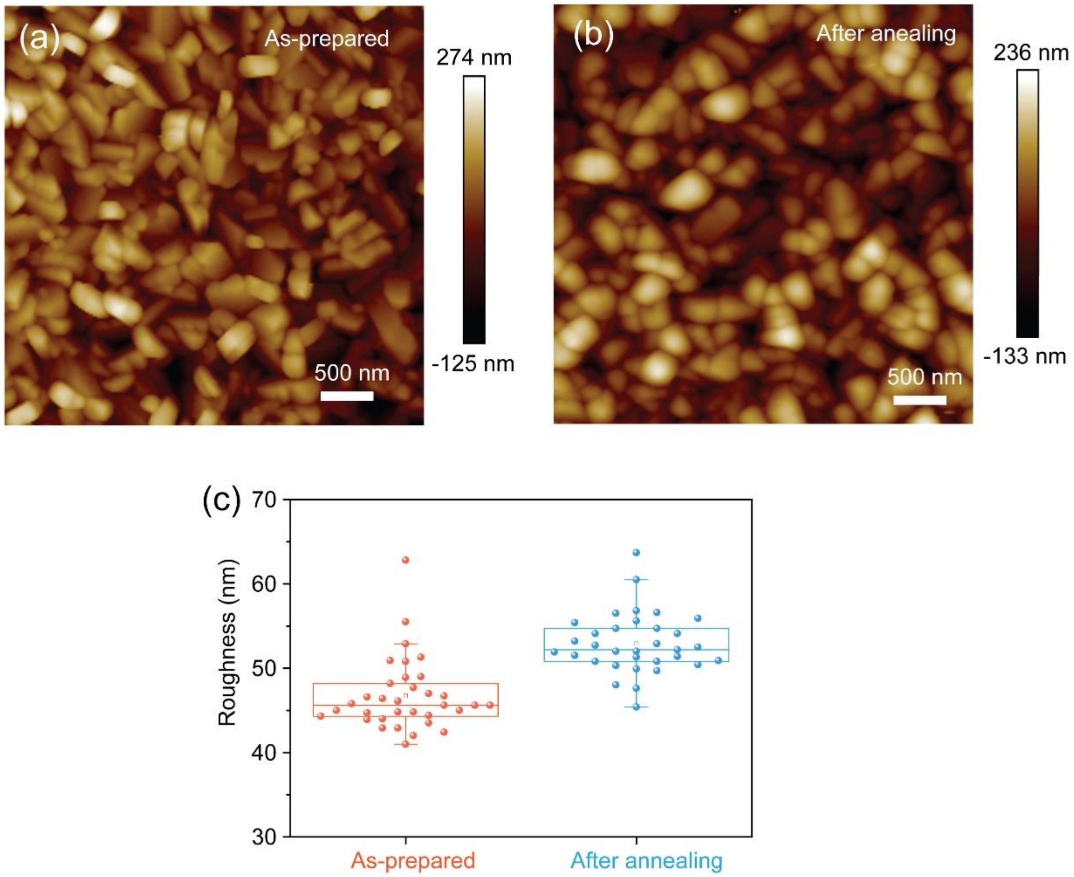


**Supplementary Figure 9 | Characterization of as-prepared GeSe film and the same film after annealed at 400°C.** AFM images of (a) as-prepared GeSe film and (b) the same film after annealed at 400°C. (c) The average root mean square (RMS) roughness taken from 30 AFM images of as-prepared GeSe film and (b) the same film after annealed at 400°C.

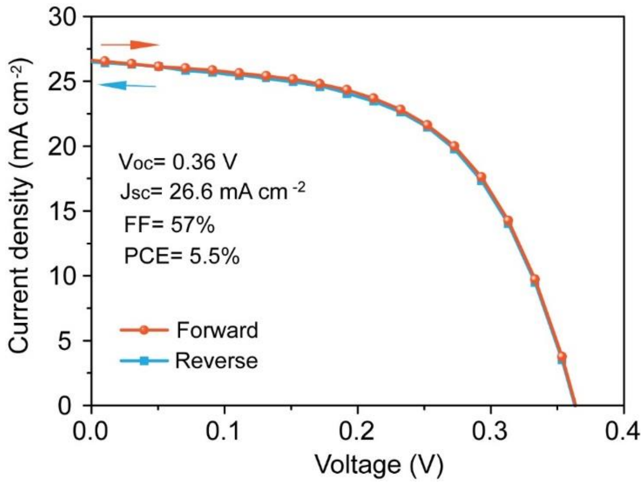


**Supplementary Figure 10 | *J-V* curves of the best-performing GeSe solar cell measured by forward and reverse scans.**

| Newport | | **Technology and Application Center**<br>**PV Lab**<br>Newport Calibration Cert. # 1896 |
|---|---|---|

**DUT S/N:** 20171205-1 D6
**Newport Calibration #: 1896**
**Manufacturer:** Institute of Chemistry, Chinese Academy of Sciences (ICCAS)
**Material:** Germanium Monoselenide (GeSe)
**Temperature Sensor:** TC-K, DUT Temperature: 25.0 ± 0.6 °C
**Environmental conditions at the time of calibration:** Temperature: 24 ± 3 °C; Humidity: 40 ± 10 %

The above DUT has been tested using the following methods to meet the ISO 17025 Standard by the PV Lab at Newport Corporation. Quoted uncertainties are expanded using a coverage factor of $k = 2$ and expressed with an approximately 95% level of confidence. Measurement of total irradiance is traceable to the World Radiometric Reference (WRR) and all other measurements and uncertainties are traceable to either NIST or CNRC and the International System of Units (SI). The performance parameters reported in this certificate apply only at the time of the test, and do not imply future performance. This certificate to be reproduced in part only with written permission from the Newport PV Laboratory. Performance parameters were determined via an IV sweep at 1.2 V/s and do not necessarily represent device performance under stabilized conditions. Cell was apertured with a thin metal mask (designated area).

| | | | | | | | | | | | |
|---|---|---|---|---|---|---|---|---|---|---|---|
| Efficency [%] | 5.20 | ± | 0.14 | V_oc [V] | 0.3756 | ± | 0.0026 | I_sc [A] | 0.000968 | ± | 0.000020 |
| P_max [mW] | 0.205 | ± | 0.005 | V_max [V] | 0.2702 | ± | 0.0030 | I_max [A] | 0.000757 | ± | 0.000016 |
| FF [%] | 56.3 | ± | 0.3 | Area [cm^2] | 0.0393 | ± | 0.0002 | M | 1.007 | ± | 0.015 |

**Methods:**
I-V: ASTM E948-16 *Standard Test Method for Electrical Performance of Photovoltaic Cells Using Reference Cells Under Simulated Sunlight*
QE: ASTM E1021-15 *Standard Test Method for Spectral Responsivity Measurements of Photovoltaic Devices*

**Standard Reporting Conditions:**
Spectrum: AM1.5-G (ASTM G173-03/IEC 60904-3 ed. 2)
1000.0 W/m $^2$ at 25.0°C

**Secondary Reference Cell:**
Device S/N: 10510-0054
Device Material: mono-Si
Window Material: fused silica
Certification: National Renewable Energy Laboratory
A2LA accreditation certificate # 2236.01
ISO Tracking #: 1936
Certified short circuit current ($I_{sc}$) under standard reporting conditions (SRC): 124.4mA
Calibration due date: 29-Sep-19

**Solar Simulator:**
Spectrum: Newport Corporation filename *Sol3A_Spectroradiometer_Scan_0199.xls*
Total irradiance: 1000 W/m$^2$ based on $I_{sc}$ of the above Secondary Reference Cell

**Quantum Efficiency for DUT:**
Newport Corporation filename *QE 1896, 20171205-1 D6, WLB.log*
Spectral mismatch correction factor: M = 1.007 ± 0.015

**DUT Calibration Procedures:**
Newport Corporation document WI1 (EQE).docx
Newport Corporation document Area Measurement WI2 (Area).docx
Newport Corporation document WI3 (IV.Sweep).docx

| Cal Cert V1.8 | Issue Date: May 17, 2018 | Page 2 of 2 |
|---|---|---|
| | Reviewed and Approved by: Geoffrey Wicks | |

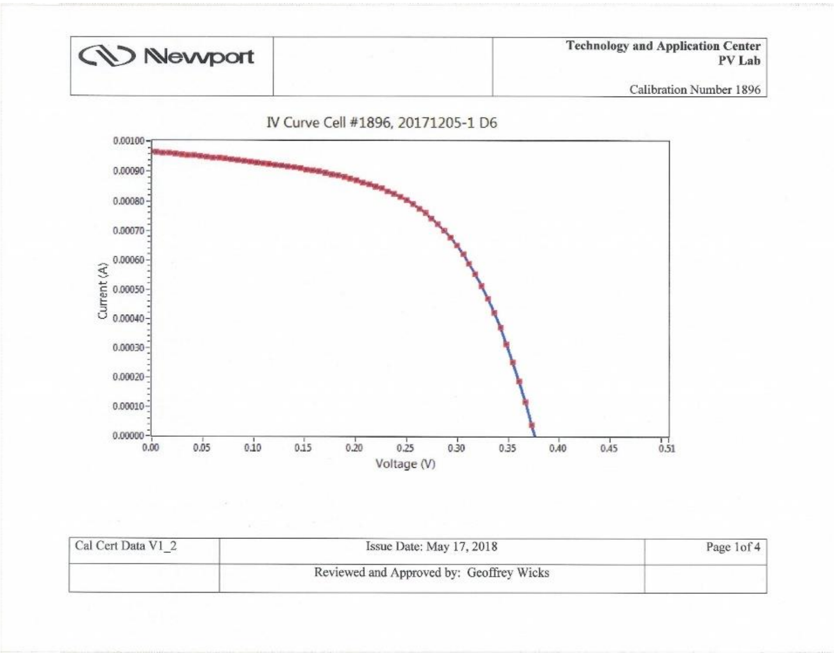

| Newport | | Technology and Application Center<br>PV Lab<br>Calibration Number 1896 |
|---|---|---|



| Cal Cert Data V1_2 | Issue Date: May 17, 2018 | Page 1of 4 |
|---|---|---|
| | Reviewed and Approved by: Geoffrey Wicks | |

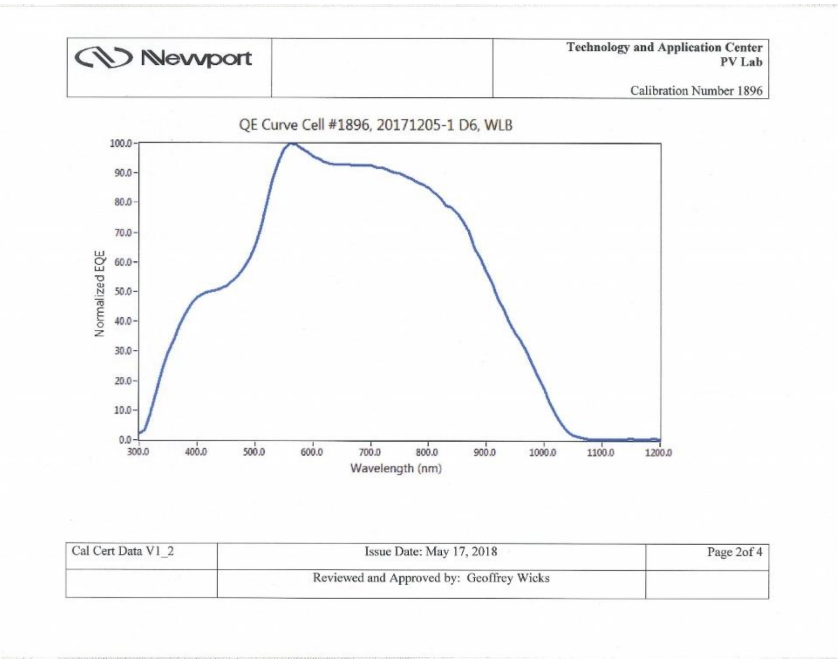

| Newport | | Technology and Application Center<br>PV Lab<br>Calibration Number 1896 |
|---|---|---|



| Cal Cert Data V1_2 | Issue Date: May 17, 2018 | Page 2of 4 |
|---|---|---|
| | Reviewed and Approved by: Geoffrey Wicks | |

**Supplementary Figure 11 | Certification results of GeSe solar cell at Newport, USA. Permission to use granted by Newport Corporation. All rights reserved.**

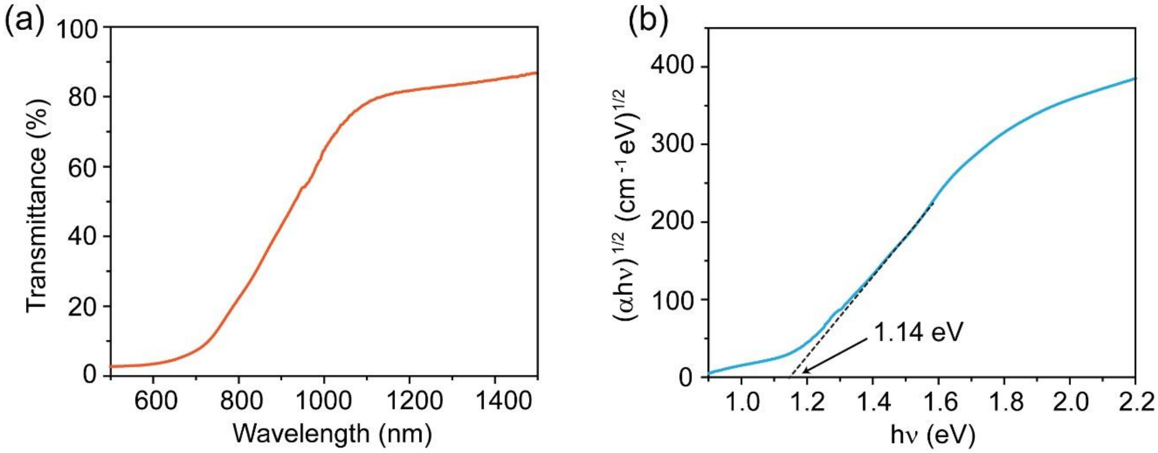


**Supplementary Figure 12 | Optical characterization of GeSe films.** (a) Transmittance spectrum of GeSe film prepared by rapid thermal sublimation (RTS) method; (b) Tauc plot (n = 1/2, indirect) for GeSe film.

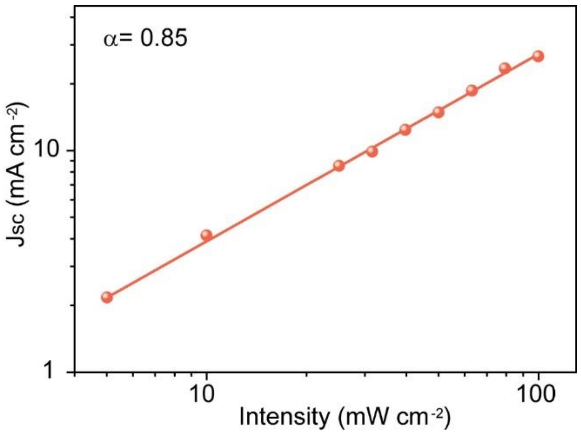


**Supplementary Figure 13 | Characterization of the performance of GeSe solar cells under different light intensities.** Light intensity-dependent $J_{sc}$ of GeSe solar cells. Neutral-density filters (THORLABS) were used to adjust the light intensity.

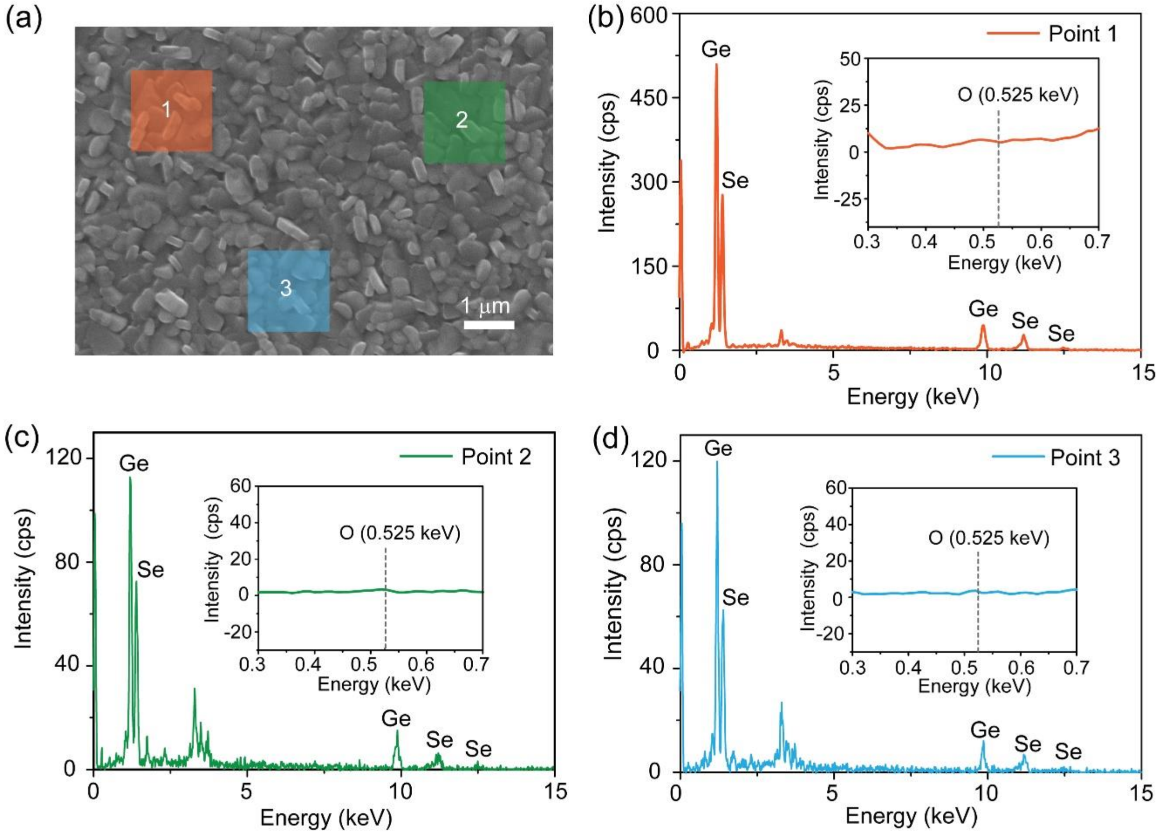


**Supplementary Figure 14 | Characterization of the GeSe film after temperature-dependent XRD measurements.** (a) Top-view SEM image of GeSe film after temperature-dependent XRD measurements. EDS spectra of GeSe film after temperature-dependent XRD measurements in the points (b) 1, (c) 2, and (d) 3, respectively. Note that no O signal is observed in all of three magnified EDS patterns (insets in Supplementary Figure 14b-d).

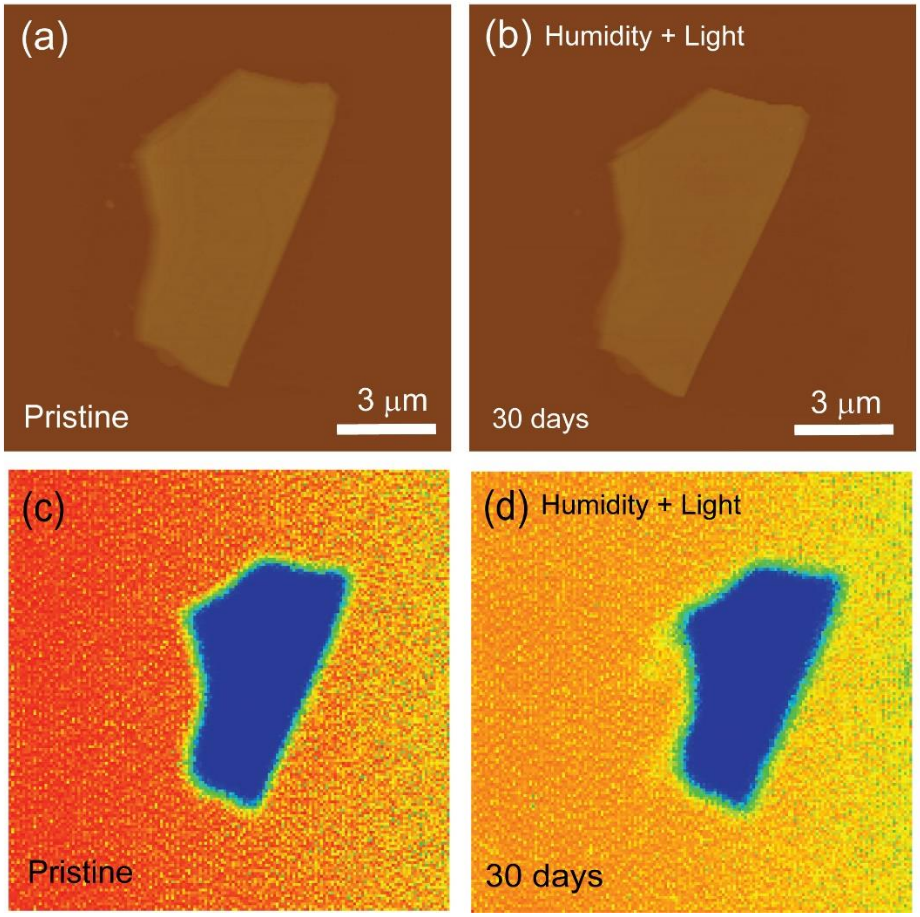


**Supplementary Figure 15 | Investigation of humidity and light stability of GeSe.** The GeSe thin flake was mechanically exfoliated from bulk crystal onto the $SiO_2$/Si substrate through scotch tape technique. AFM images of (a) pristine GeSe thin flake and (b) the sample after 30 days aging under 85% humidity and continuous 1 sun illumination. Spatial Raman mapping images of (c) pristine GeSe thin flake and (d) the aging sample for 30 days under 85% humidity and continuous 1 sun illumination. The morphology and Raman mapping of GeSe thin flake displayed no change after 30 days aging under 85% humidity and continuous 1 sun illumination, demonstrating the excellent humidity and light stability of GeSe.

**Supplementary Table 1 | Photovoltaic parameters of GeSe solar cell measured under the different intensities of simulated AM1.5G illumination.**

| **Light intensity [mW cm$^{-2}$]** | $V_{oc}$ **[V]** | $J_{sc}$ **[mA cm$^{-2}$]** | **FF [%]** | **PCE [%]** |
|---|---|---|---|---|
| **100** | 0.354 | 26.6 | 53.1 | 5.0 |
| **50** | 0.327 | 14.9 | 54.3 | 5.3 |
| **10** | 0.28 | 4.14 | 53.9 | 6.3 |
| **1** | 0.202 | 0.84 | 50.6 | 8.6 |

## Supplementary References


1. Zakutayev, A. *et al.* Defect tolerant semiconductors for solar energy conversion. *J. Phys. Chem. Lett.* **5**, 1117-1125 (2014).
2. Ganose, A. M., Savory, C. N. & Scanlon, D. O. Beyond methylammonium lead iodide: prospects for the emergent field of $ns^2$ containing solar absorbers. *Chem. Commun.* **53**, 20-44 (2017).
3. Kang, J. & Wang, L.-W. High defect tolerance in lead halide perovskite $CsPbBr_3$. *J. Phys. Chem. Lett.* **8**, 489-493 (2017).
4. Brandt, R. E., Stevanović, V., Ginley D. S. & Buonassisi T. Identifying defect-tolerant semiconductors with high minority-carrier lifetimes: beyond hybrid lead halide perovskites. *MRS Commun.* **5**, 265-275 (2015).
5. Brandt, R. E. *et al.* Searching for “defect-tolerant” photovoltaic materials: combined theoretical and experimental screening. *Chem. Mater.* **29**, 4667-4674 (2017).
6. Walsh, A., Payne, D. J., Egdell, R. G. & Watson, G. W. Stereochemistry of post-transition metal oxides: revision of the classical lone pair model. *Chem. Soc. Rev.* **40**, 4455-4463 (2011).
7. Kurchin, R. C., Gorai, P., Buonassisi, T. & Stevanović, V. Structural and chemical features giving rise to defect tolerance of binary semiconductors. *Chem. Mater.* **30**, 5583-5592 (2018).
