# Supplementary material for: An antibonding valence band maximum enables defect-tolerant and stable GeSe photovoltaics
Source: Nat Commun. 2021 Jan 28;12:670. doi: 10.1038/s41467-021-20955-5 (PMC7844217; doi:10.1038/s41467-021-20955-5)
Supplement: Supplementary file 4 — Solar Cells Reporting Summary [file 41467_2021_20955_MOESM4_ESM.pdf]

nature research

Corresponding author(s): Edward H. Sargent

Solar Cells Reporting Summary

Nature Research wishes to improve the reproducibility of the work that we publish. This form is intended for publication with all accepted papers reporting the characterization of photovoltaic devices and provides structure for consistency and transparency in reporting. Some list items might not apply to an individual manuscript, but all fields must be completed for clarity.

For further information on Nature Research policies, including our data availability policy, see Authors & Referees.

Experimental design

Please check: are the following details reported in the manuscript?

1. Dimensions

Item	Yes/No	Details
Area of the tested solar cells	☒ Yes ☐ No	0.09 cm2 (Methods)
Method used to determine the device area	☒ Yes ☐ No	The device area is determined by the aperture shade mask (Methods)

2. Current-voltage characterization

Item	Yes/No	Details
Current density-voltage (J-V) plots in both forward and backward direction	☒ Yes ☐ No	Supplementary Fig. 10
Voltage scan conditions<br>*For instance: scan direction, speed, dwell times*	☒ Yes ☐ No	JV curves were measured with a scanning rate of 100 mV/s (voltage step of 20 mV and delay time of 200 ms) (Methods)
Test environment<br>*For instance: characterization temperature, in air or in glove box*	☒ Yes ☐ No	Performance measurements were carried out in air at room temperature (Methods)
Protocol for preconditioning of the device before its characterization	☒ Yes ☐ No	No preconditioning was used.
Stability of the J-V characteristic<br>*Verified with time evolution of the maximum power point or with the photocurrent at maximum power point; see ref. 7 for details.*	☒ Yes ☐ No	Maximum power point tracking (Fig. 4b)

3. Hysteresis or any other unusual behaviour

Item	Yes/No	Details
Description of the unusual behaviour observed during the characterization	☒ Yes ☐ No	There is no hysteresis
Related experimental data	☒ Yes ☐ No	Supplementary Fig. 10

4. Efficiency

Item	Yes/No	Details
External quantum efficiency (EQE) or incident photons to current efficiency (IPCE)	☒ Yes ☐ No	Fig. 3e
A comparison between the integrated response under the standard reference spectrum and the response measure under the simulator	☒ Yes ☐ No	Fig. 3e
For tandem solar cells, the bias illumination and bias voltage used for each subcell	☐ Yes ☒ No	All devices are single-junction solar cells (Methods)

5. Calibration

Item	Yes/No	Details
Light source and reference cell or sensor used for the characterization	☒ Yes ☐ No	Described in the Method section
Confirmation that the reference cell was calibrated and certified	☒ Yes ☐ No	The light intensity was calibrated by reference solar cell by Newport.

Calculation of spectral mismatch between the reference cell and the devices under test

☒ Yes ☐ No — Described in the Method section

6. Mask/aperture

Size of the mask/aperture used during testing

☒ Yes ☐ No — 0.09 cm2 (Methods)

Variation of the measured short-circuit current density with the mask/aperture area

☐ Yes ☒ No — We always tested device performance at the same aperture area.

7. Performance certification

Identity of the independent certification laboratory that confirmed the photovoltaic performance

☒ Yes ☐ No — Certificated by Newport

A copy of any certificate(s)
Provide in Supplementary Information

☒ Yes ☐ No — Supplementary Fig. 11

8. Statistics

Number of solar cells tested

☒ Yes ☐ No — Described in Fig. 3c

Statistical analysis of the device performance

☒ Yes ☐ No — Described in Fig. 3c

9. Long-term stability analysis

Type of analysis, bias conditions and environmental conditions
For instance: illumination type, temperature, atmosphere humidity, encapsulation method, preconditioning temperature

☒ Yes ☐ No — Depicted in Figures 4a, 4b, 4c, 4d and the Method section
